# The prognostic value of AGR2 expression in solid tumours: a systematic review and meta-analysis

**DOI:** 10.1038/s41598-017-15757-z

**Published:** 2017-11-14

**Authors:** Shao-bo Tian, Kai-xiong Tao, Jia Hu, Zhi-bo Liu, Xue-liang Ding, Ya-nan Chu, Jin-yuan Cui, Xiao-ming Shuai, Jin-bo Gao, Kai-lin Cai, Ji-liang Wang, Guo-bin Wang, Lin Wang, Zheng Wang

**Affiliations:** 1 Department of Gastrointestinal Surgery, Union Hospital, Tongji Medical College, Huazhong University of Science and Technology, Wuhan 430022, China; 2 Research Centre for Tissue Engineering and Regenerative Medicine, Union Hospital, Tongji Medical College, Huazhong University of Science and Technology, Wuhan 430022, China; 3 Department of Clinical Laboratory, Union Hospital, Tongji Medical College, Huazhong University of Science and Technology, Wuhan 430022, China

## Abstract

The prognostic value of anterior gradient-2 (AGR2) in tumours remains inconclusive. Here, we systematically reviewed the literature evidence and assessed the association between AGR2 expression and prognosis in solid tumours. The primary outcomes were overall survival (OS), disease-specific survival (DSS), and disease-free survival (DFS)/recurrence-free survival (RFS)/progression-free survival (PFS). All analyses were performed by STATA 12.0, with the hazard ratio (HR) or odds ratios (OR), and 95% confidence interval (CI) as the effect size estimate. A total of 20 studies containing 3285 cases were included. Pooled analyses revealed that AGR2 overexpression had an unfavourable impact on OS (HR 1.93, 95% CI 1.32–2.81) and time to tumour progression (TTP) (DFS/RFS/PFS) (HR 1.60 95% CI 1.06–2.40) in solid tumour patients. Subgroup analyses indicated that AGR2 overexpression in breast cancer patients was significantly associated with poor OS (HR 3.02, 95% CI 1.03–8.81) and TTP (HR 1.93, 95% CI 1.17–3.20). Excluding breast cancer, AGR2 overexpression was also found to have a significant correlation with poor OS in the remaining solid tumour patients (HR 1.51, 95% CI 1.04–2.19). Overall, AGR2 might be a potential biomarker to predict prognosis in solid tumour patients.

## Introduction

The human anterior gradient-2 (AGR2), a homologue of xenopus anterior gradient-2 (XAG-2) of *Xenopus laevis* ^1^, is a member of the protein disulfide isomerase (PDI) gene family. AGR2 protein weighs 19 kDa containing a short N-terminal signal peptide and a C-terminal endoplasmic reticulum retention sequence (KTEL)^2^. AGR2, physiologically localized in endoplasmic reticulum (ER), has emerged as a critical modulator of ER homeostasis^3,4^. A growing body of evidence supports a functional role of AGR2 in a variety of cellular functions, such as cell migration, differentiation and proliferation^5^.

Since AGR2 was found to be as a pro-oncogenic protein that attenuates p53 activity in 2004^6^, AGR2’s molecular role and its clinical relevance have been increasingly investigated in cancers, including breast^7,8^, lung^9^, ovarian^10^, prostate^11^, pancreatic cancer^12^, and colorectal carcinomas^13^. Although high AGR2 expression in breast and lung cancer was reportedly correlated with poor clinical prognosis^14,15^, some studies suggested the otherwise, thus resulting in a controversy^9,16^. Moreover, in other types of cancer, such as prostate cancer, ovarian cancer and colorectal cancer, the prognostic value of AGR2 remains largely inconclusive^17–19^. Aiming to explore the prognostic value of AGR2 in these solid tumours, we conducted this comprehensive meta-analysis.

## Results

### Description of the selected studies

**Figure 1:**
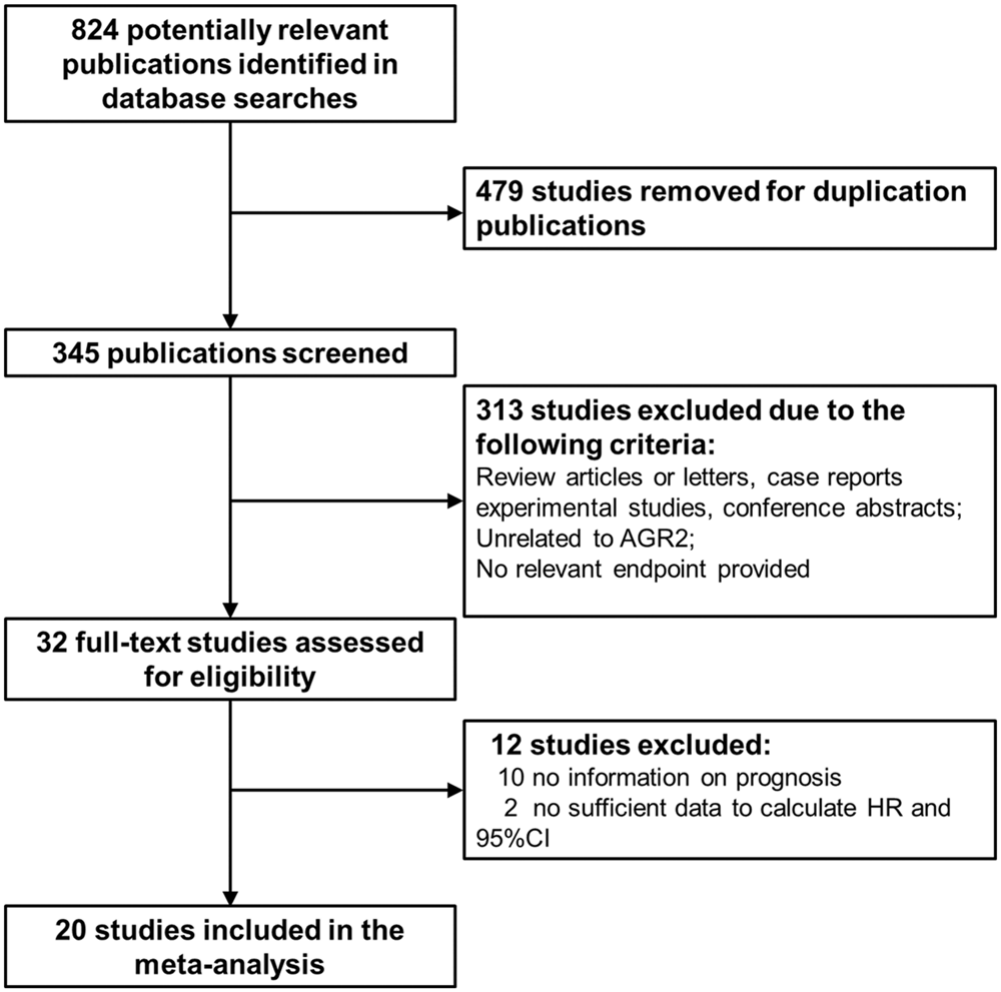
Flow diagram of the selection process.

A total of 824 studies were initially identified using our search strategy from PubMed, Embase, and Web of Science database (Fig. 1). 791 studies were discarded because of either duplication (479) or irrelevance (312). Out of 32 studies eligible for further assessment, 12 studies were excluded with 10 of them lacking prognosis data and the rest of two studies having insufficient data for estimating HR with 95% CI. Therefore, 20 studies (19 in English and 1 in Chinese) with a total number of 3285 patients were used for analysing the relationship between AGR2 expression and solid tumour patients’ prognosis. These 20 included studies were of high methodological quality with their Newcastle-Ottawa Scale (NOS) scores ranging from 6 to 9.

The main features of the 20 eligible studies^9,14–32^ were extracted and summarized in Table 1. These studies were conducted in nine countries with 13 studies originated from Europe (5 from Germany; 4 from UK; 3 from Czech Republic; 1 from Spain), 5 from Asia (2 from China; 2 from Japan; 1 from Singapore), 1 from USA, and 1 from Australia. These studies were published between 2005 and 2016. The median follow-up time of the 14 studies with definite follow-up duration was 53 months (ranging from 23 to 192). As for cancer types, breast cancer was the most frequent cancer type (n = 9), followed by lung cancer (n = 4), prostate cancer (n = 2), ovarian cancer (n = 2), colorectal cancer (n = 2), and gastric cancer (n = 1). Given that DFS /RFS /PFS are similar outcome endpoints, we thus used the time to tumour progression (TTP) to represent these three survival parameters in our meta-analysis^33^. TTP referred to the length of time from the date of diagnosis or the treatment starting date to the date when the disease condition started to progresses again or metastasis was detected. In these studies, quantitative reverse transcription polymerase chain reaction (qRT-PCR) (n = 2) or immunohistochemistry (IHC) staining (n = 16) was used to detect AGR2 expression in tumour tissue, while ELISA (n = 1) or qRT-PCR (n = 1) was employed to measure AGR2 expression in serum samples. The mean expression level (Table 1) was the most frequently used cut-off value for AGR2 positive expression across these studies.

**Table 1:** Characteristic of the included studies.

**Cancer type**	**Study**	**Country**	**Case**	**Age median**	**Test method**	**location**	**Cut-off value**	**Follow-up time (range) months**	**Outcome endpoints**	**NOS**
Prostate cancer	Kristiansen *et al*.^17^	Germany	91	63	IHC	tumour	Positive:weak & intermediate & strong	30.5(2–84)	DFS	8
							Negative:complete absence of immunoreactivity			
Breast cancer	Fritzsche *et al*.^20^	Germany	155	59	IHC	tumour	Positive (score = 1–12)	75(1–162)	OS,DFS	8
							Negative(score = 0) (Range of 0–12)			
Breast cancer	Innes *et al*.^21^	UK	225	64	IHC	tumour	Positive: ≥ 1% carcinoma cells stained	85.9(0.1–212)	OS	8
							Negative: < 1% carcinoma cells stained			
Lung cancer	Fritzsche *et al*.^9^	Germany	77	62	IHC	tumour	Positive: score1&2	23(0–92)	DSS	8
							Negative:score 0 (Range of 0–2)			
Prostate cancer	Zhang *et al*.^22^	UK	65	73	IHC	tumour	Positive: (2–16)	NA	OS	7
							Negative:(1) (Range of 1–16)			
Breast cancer	Wu *et al*.^23^	China	72	50	IHC	tumour	Positive:stained in the cytoplasm, yellow or brown particles	60(8–64)	OS	8
							Negative:complete absence			
Breast cancer	Barraclough *et al*.^14^	UK	315	57	IHC	tumour	Positive: ≥ 1% carcinoma cells stained	192(168–240)	OS	8
							Negative: < 1% carcinoma cells stained			
Breast cancer	Hrstka *et al*.^24^	Czech Republic	78	NA	QRT-PCR	tumour	High: > the mean expression levels	48	DFS	7
							Low: ≤ the mean expression levels			
Lung cancer	Chung *et al*.^25^	Japan	111	68	ELISA	serum	Positive: > 2.6ng/ml	36(4–77)	OS,DFS	8
							Negative: < 2.6ng/ml			
Lung cancer	Chung *et al*.^27^	Japan	212	67	IHC	tumour	Positive: > 50% carcinoma cells stained	24(3–61)	DSS	8
							Negative: < 1% carcinoma cells stained			
Breast cancer	Rudland *et al*.^26^	UK	137	60.3	IHC	tumour	Positive: ≥ 1% carcinoma cells stained	192(168–240)	OS	8
							Negative: < 1% carcinoma cells stained			
Colorectal Cancer	Valladares-Ayerbes *et al*.^29^,	Spain	54	62.7	QRT-PCR	serum	High: > the mean expression levels	58(17–84)	OS,PFS	8
							Low: ≤ the mean expression levels			
Ovarian cancer	Darb-Esfahani *et al*.^28^,	Germany	124	NA	IHC	tumour	High: > 50% carcinoma cells stained	45 (2.5–162.3)	OS,PFS	8
							Low: ≤ 50% carcinoma cells stained			
Ovarian cancer	Armes *et al*.^18^,	Australia	59	NA	IHC	tumour	Positive: > 50% carcinoma cells stained	NA	DFS	7
							Negative: ≤ 50% carcinoma cells stained			
Breast Cancer	Hrstka *et al*.^24^,	Czech Republic	61	79	QRT-PCR	tumour	High: > the mean expression levels	NA	OS,PFS	8
							Low: ≤ the mean expression levels			
Colorectal Cancer	Riener *et al*.^19^,	Germany	432	72	IHC	tumour	High:score 2 or 3	42 (1–153)	OS	9
							Low:score 0 or 1 (Range of 0–3)			
Lung cancer	Alavi *et al*.^15^,	USA	155	NA	IHC	tumour	High: > the mean expression levels	NA	OS	7
							Low: ≤ the mean expression levels			
Breast Cancer	Hrstka *et al*.^16^,	Czech Republic	234	NA	IHC	tumour	High: > the mean expression levels	NA	RFS	7
							Low: ≤ the mean expression levels			
Breast Cancer	Lacambra *et al*.^31^,	Singapore	400	53.9	IHC	tumour	Positive: ≥ 5% of cells with strong to moderate cytoplasmic staining	61.3 (3–210)	DFS	8
							Negative: < 5% of cells with strong to moderate cytoplasmic staining			
Gastric cancer	Zhang *et al*.^22^,	China	228	NA	IHC	tumour	High:the product of the staining intensity and proportion of stained tumor cells scores ≥ 4	NA	OS	7
							Low:the product of the staining intensity and proportion of stained tumor cells scores ≤ 3			

Abbreviations: NA: not available; IHC: Immunohistochemistry; QRT-PCR: Quantitative Real Time Polymerase Chain Reaction; NOS: Newcastle-Ottawa Scale; OS: overall survival; DSS: disease specific survival; DFS: disease free survival; RFS: recurrence free surviv.

### Impact of high AGR2 expression on cancer prognosis

**Figure 2:**
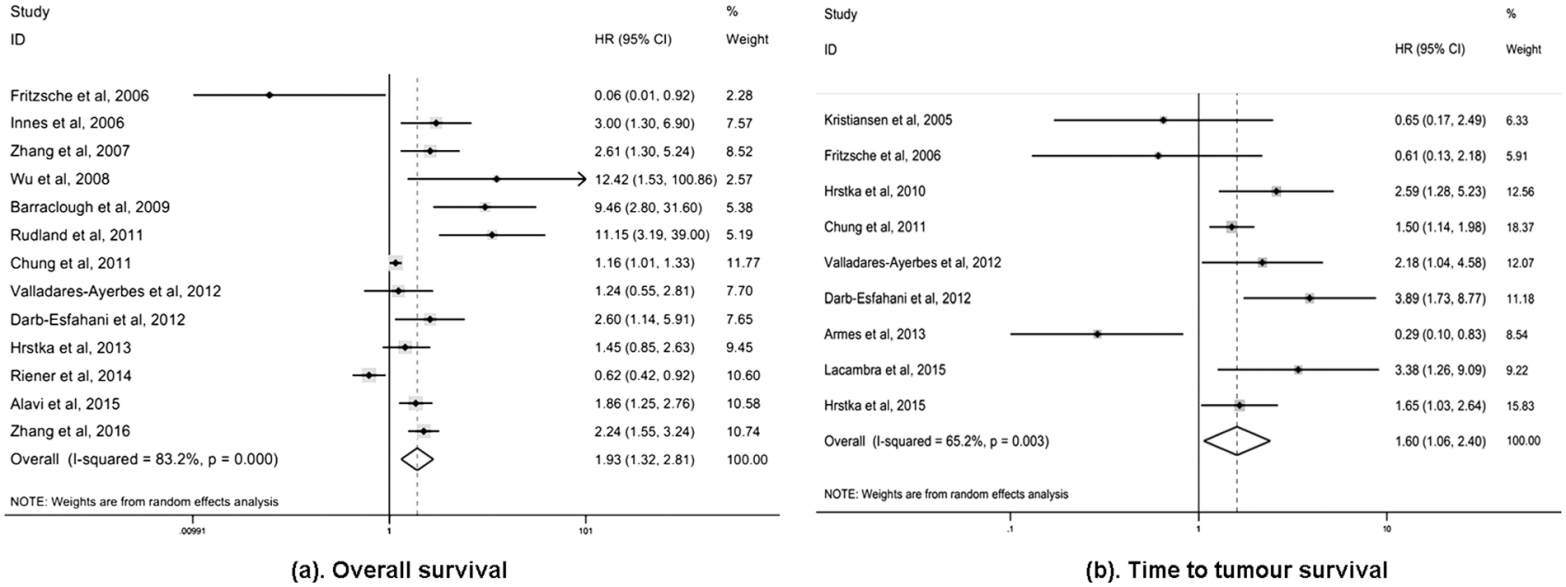
Meta**-**analysis of impact of AGR2 expression on prognosis of patients with solid tumours. Forest plot of HRs for the correlation between AGR2 overexpression and OS **(a)** and TTP **(b)** in solid tumour patients.

Concerning the survival outcomes in patients with solid malignancies, 13 studies evaluated the relationship between AGR2 expression and OS, while 8 studies analysed the association of AGR2 expression with TTP and two studies with DSS. OS was indicated by the percentage of patients who remained alive at a given time point. DSS was indicated using the percentage of subjects who survived a particular disease for a defined period of time^33^. The estimated pooled HRs showed that compared with AGR2 low/ negative expression, AGR2 overexpression/ positivity was highly related to poor OS (HR 1.93, 95% CI 1.32–2.81) and poor TTP (HR 1.60, 95% CI 1.06–2.40) of solid tumour patients (Fig. 2). However, no association was found between AGR2 overexpression and DSS (HR 0.36, 95% CI 0.06–2.14). These pooled analyses were conducted using the random effects model, in which significant heterogeneity of the included studies on OS (*I* ^2^ = 83.2%, *P* = 0.000), TTP (*I* ^2^ = 65.2%, *P* = 0.003) and DSS (*I* ^2^ = 77.6%, *P* = 0.035) was observed, indicating that the choice of this model was appropriate (Table 2).

**Table 2:** Hazard ratio for the association between AGR2 overexpression and solid tumours prognosis.

**Stratified analysis**	**Effect size**	**NO. of study**	**References**	**Cases**	**Pooled HR (95% CI)**	***P*** **value**	**Heterogeneity**	
							***I*** ^**2**^ **(%)**	***p*** **value**
**All studies**								
**OS**	OS	13	Fritzsche *et al*.^20^; Innes *et al*.^21^; Zhang *et al*.^22^; Wu *et al*.^23^; Barraclough *et al*.^14^; Chung *et al*.^25^; Rudland *et al*.^26^; Darb-Esfahani *et al*.^28^; Valladares-Ayerbes *et al*.^29^ Hrstka *et al*.^30^; Riener *et al*.^19^; Alavi *et al*.^15^;	2164	**1.93 (1.32–2.81)**	**0.001**	**83.2**	**0.000**
DSS	DSS	2	Fritzsche *et al*.^9^; Chung *et al*.^27^	289	0.36 (0.06–2.14)	0.261	77.6	0.035
**TTP**	TTP	9	Kristiansen *et al*.^17^; Fritzsche *et al*.^20^; Armes *et al*.^18^; Hrstka *et al*.^24^; Chung *et al*.^25^; Darb-Esfahani *et al*.^28^; Valladares-Ayerbes *et al*.^29^; Hrstka *et al*.^30^; Lacambra *et al*.^31^	1306	**1.60 (1.06–2.40)**	**0.007**	**65.2**	**0.003**
**Study location**								
Europe	OS	9	Fritzsche *et al*.^20^; Innes *et al*.^21^; Zhang *et al*.^22^; Barraclough *et al*.^14^; Rudland *et al*.^26^; Darb-Esfahani *et al*.^28^; Hrstka *et al*.^16^; Riener *et al*.^19^	1598	1.96 (0.99–3.85)	0.052	84.8	0.000
	DSS	1	Fritzsche *et al*.^9^	77	0.81 (0.35–1.89)	0.624		
	TTP	6	Kristiansen *et al*.^17^; Fritzsche *et al*.^20^; Hrstka *et al*.^24^; Darb-Esfahani *et al*.^28^; Valladares-Ayerbes *et al*.^29^; Hrstka *et al*.^30^	736	**1.88 (1.20–2.94)**	**0.000**	**45.4**	**0.103**
Asia	OS	3	Wu *et al*.^23^; Chung *et al*.^25^; Zhang *et al*.^32^	411	1.91 (0.95–3.84)	0.07	86.9	0.000
	DSS	1	Chung *et al*.^27^;	212	0.13 (0.03–0.57)	0.007	—	—
	TTP	2	Chung *et al*.^25^; Lacambra *et al*.^31^	511	1.95 (0.93–4.10)	0.076	58.5	0.121
Oceania	TTP	1	Armes *et al*.^18^	59	**0.29 (0.10–0.84)**	**0.022**	—	—
USA	OS	1	Alavi *et al*.^15^	155	**1.86 (1.25–3.31)**	**0.002**	—	—
**Cancer type**								
Breast cancer	OS	6	Fritzsche *et al*.^20^; Innes *et al*.^21^; Wu *et al*.^23^; Barraclough *et al*.^14^; Rudland *et al*.^26^; Hrstka *et al*.^16^	995	**3.02 (1.03–8.81)**	**0.044**	**81.2**	**0.000**
	TTP	4	Fritzsche *et al*.^20^; Hrstka *et al*.^24^; Hrstka *et al*.^30^; Lacambra *et al*.^31^	867	**1.93 (1.17–3.20)**	**0.000**	**38.7**	**0.180**
Lung cancer	OS	2	Chung *et al*.^25^; Alavi *et al*.^15^	266	1.41 (0.90–2.23)	0.137	79.5	0.027
	DSS	2	Fritzsche *et al*.^9^; Chung *et al*.^27^	289	0.36 (0.06–2.14)	0.261	77.6	0.035
	TTP	1	Chung *et al*.^25^	111	**1.50 (1.14–1.98)**	**0.004**	—	—
Prostate cancer	OS	1	Zhang *et al*.^22^	65	**2.61 (1.30–5.24)**	**0.044**	—	—
	TTP	1	Kristiansen *et al*.^17^	91	0.65 (0.17–2.49)	0.529	—	—
Ovarian cancer	OS	1	Darb-Esfahani *et al*.^28^	124	**2.60 (1.14–5.92)**	**0.023**	—	—
	TTP	2	Darb-Esfahani *et al*.^28^; Armes *et al*.^18^	183	1.09 (0.09–13.84)	0.949	93.1	0.000
Colorectal Cancer	OS	2	Valladares-Ayerbes *et al*.^29^; Riener *et al*.^19^;	486	0.80 (0.41–1.53)	0.495	55.6	0.133
	TTP	1	Valladares-Ayerbes *et al*.^29^	54	**2.18 (1.04–4.57)**	**0.039**	—	—
Gastric cancer	OS	1	Zhang *et al*.^32^	228	**2.24 (1.55–3.24)**	**0.000**	—	—

Abbreviations: No.:number; HR: hazard ratio; CI:confidence interval; OS:overall survival; DSS: disease specific survival; TTP:time to tumor progression.

### Subgroup analysis

To identify the contributing factors underlying heterogeneity, subgroup analyses by cancer types and study location were conducted (Table 2). If only one study provided relevant data on the correlation of AGR2 overexpression with outcome endpoints, the subgroup analysis was not performed. The subgroup analysis by study location indicated that 13 studies reporting OS were stratified into Europe (n = 9), Asia (n = 3), and USA (n = 1); 9 studies focused on TTP were stratified into Europe (n = 6), Asia (n = 2), and Oceania (n = 1). According to the different study locations, subgroup analyses did not reveal any significant correlation between AGR2 overexpression and the OS of solid tumour patients (in Europe: HR1.96, 95% CI 0.99–3.85; in Asia: HR1.91 95% CI 0.95–3.84). Furthermore, the heterogeneity could not be reduced by location stratification either (in Europe: *I* ^2^ = 84.8%, *P* = 0.000; in Asia: *I* ^2^ = 86.9%, *P* = 0.000). However, we observed a significant association between AGR2 overexpression and poor TTP (HR1.88, 95% CI 1.20–2.94) with no obvious heterogeneity (*I* ^2^ = 45.4%, *P* = 0.103) in the studies from Europe, suggesting that the study location might partially account for the heterogeneity among the studies on TTP.

**Figure 3:**
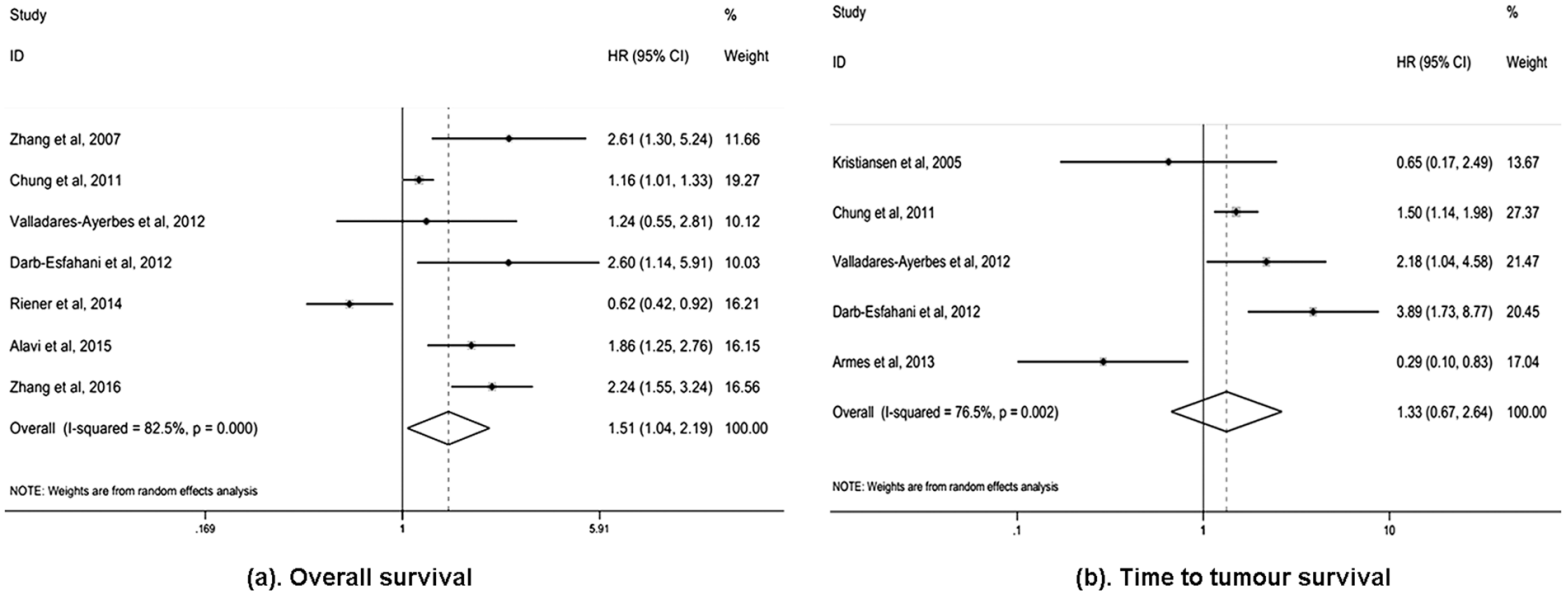
Meta**-**analysis of impact of AGR2 expression on prognosis of the solid tumours group (excluding the breast cancers). Forest plot of HRs for the correlation between AGR2 overexpression and OS (**a**) and TTP (**b**) in solid tumour patients.

**Figure 4:**
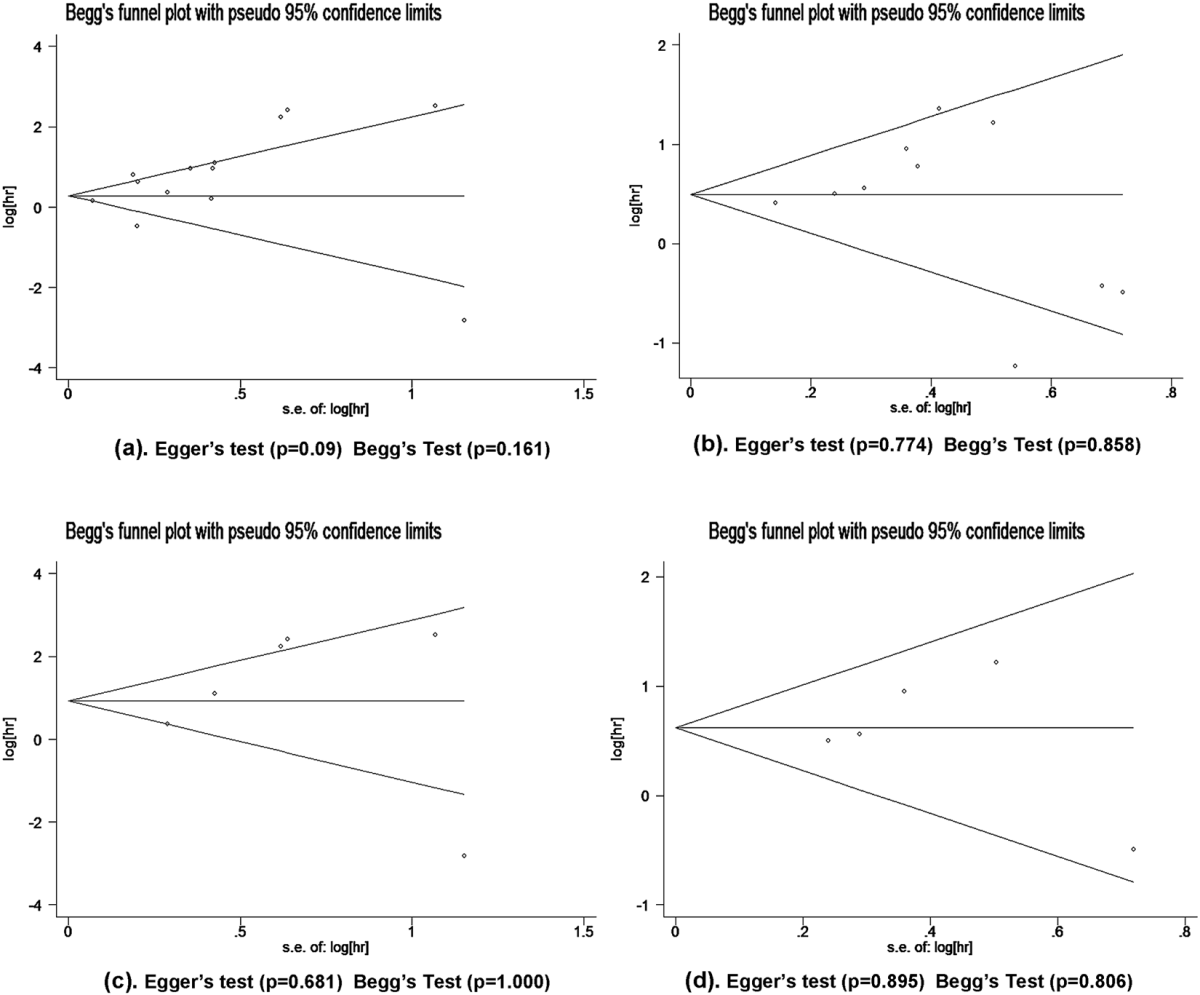
Funnel plot of studies used in the analysis of AGR2 expression and the prognosis of patients with solid tumours. (**a**) OS for solid tumour patients. (**b**) TTP for solid tumour patients. **(c)** OS for breast cancer patients. (**d**)TTP for breast cancer patients.

Notably, in breast cancer patients, AGR2 overexpression could predict poor outcomes (OS: HR 3.02, 95% CI 1.03–8.81; TTP: HR 1.93, 95% CI 1.17–3.20), which was not observed in lung cancer, ovarian cancer, and colorectal cancer (OS for lung cancer: HR 1.41, 95% CI 0.90–2.23; OS for colorectal cancer: HR 0.80, 95% CI 0.41–1.53; TTP for ovarian cancer: HR 1.09, 95% CI 0.09–13.84). In addition, meta-analyses in prostate cancer and gastric cancer were not conducted, because the number of studies (n = 1) was insufficient. Since nearly half of the included studies focused on breast cancer, we excluded them and performed subgroup analyses for the remaining solid tumours group. AGR2 overexpression still predicted poor OS (HR 1.93, 95% CI 1.32–2.81), but not TTP (HR 1.60 95% CI 1.06–2.40) (Fig. 3). These results demonstrate that AGR2 overexpression can be the prognostic factor for the OS of solid tumour patients. Begg’s test and Egger’s test as well as funnel plots revealed no obvious publication bias concerning OS and TTP in the subgroup analysis for breast cancer patients (Fig. 4c and d). However, high heterogeneity among breast cancer studies was identified for OS (*I* ^2^ = 81.2%, *P* = 0.000), but not for TTP (*I* ^2^ = 38.7%, *P* = 0.180). Thus, we set to address the heterogeneity for OS in breast cancer patients by further performing subgroup analyses on study location, follow-up time, estrogen receptor (ER) status, and sample size. The subgroup analysis showed an improved HR in the studies with large sample size ( ≥ 200, HR 4.88, 95% CI 1.60–14.85) and long follow-up time ( ≥ 80 months, HR 6.14, 95% CI 2.53–14.92). Moreover, the heterogeneity across the studies with large sample size (*I* ^2^ = 57.3%, *P* = 0.216) and long follow-up time (*I* ^2^ = 50.2%, *P* = 0.134) were found to be effectively reduced. The subgroup analysis on ER status also confirmed the unfavourable impact of AGR2 overexpression on the OS of ER positive breast cancer patients (HR 2.58, 95% CI 1.06–6.26). In terms of study location, no significant association between AGR2 overexpression and poor OS was identified in breast cancer studies from Europe (HR 2.48, 95% CI 0.79–7.81) (Table 3).

**Table 3:** Subgroup analyses of the association between AGR2 overexpression and OS for breast cancer patients.

**Stratified analysis**	**NO. of study**	**References**	**Cases**	**Pooled HR (95% CI)**	***P*** **value**	**Heterogeneity**	
						***I*** ^**2**^ **(%)**	***p*** **value**
Total	6	Fritzsche *et al*.^20^; Innes *et al*.^21^; Wu *et al*.^23^; Barraclough *et al*.^14^; Rudland *et al*.^26^; Hrstka *et al*.^16^	995	3.02 (1.03–8.81)	0.044	81.2	0.000
**ER status**							
Positive	3	Innes *et al*.^21^; Wu *et al*.^23^; Hrstka *et al*.^16^	388	**2.58 (1.06–6.26)**	**0.036**	**60.6**	**0.079**
Negative	3	Fritzsche *et al*.^20^; Barraclough *et al*.^14^; Rudland *et al*.^26^	607	2.26 (0.18–28.56)	0.530	88.5	0.000
**Study location**							
Europe	5	Fritzsche *et al*.^20^; Innes *et al*.^21^; Barraclough *et al*.^14^; Rudland *et al*.^26^; Hrstka *et al*.^16^	923	2.48 (0.79–7.81)	0.122	83.5	0.000
Asia	1	Wu *et al*.^23^	72	12.42 (1.53–100.84)	0.018	—	—
**Sample size**							
≥ 200	2	Innes *et al*.^21^; Barraclough *et al*.^14^	570	**4.88 (1.60–14.85)**	**0.005**	**57.3**	**0.126**
<200	4	Fritzsche *et al*.^20^; Wu *et al*.^23^; Hrstka *et al*.^16^; Rudland *et al*.^26^	425	2.08 (0.35–12.32)	0.421	85.4	0.000
**Follow-up (months)**							
≥ 80	3	Innes *et al*.^21^; Rudland *et al*.^26^; Barraclough *et al*.^14^	707	**6.14 (2.53–14.92)**	**0.000**	**50.2**	**0.134**
<80	3	Fritzsche *et al*.^20^; Wu *et al*.^23^; Hrstka *et al*.^16^	288	1.10 (0.114–10.65)	0.935	82.8	0.003

Abbreviations: No. :number; HR: hazard ratio; CI: confidence interval; OS: overall survival. ER: estrogen receptor.

### Correlation of AGR2 expression with clinicopathological features of breast cancer

To fully elucidate AGR2′s clinical relevance in breast cancer, we assessed the relationship between AGR2 overexpression and clinicopathological features of breast cancer patients. As illustrated in Table 4, AGR2 overexpression correlated positively with ER status (positive vs negative: OR 4.08, 95% CI 2.16–7.69), PgR status (positive vs negative: OR 2.87, 95% CI 2.17–3.80), and negatively with histological grade (III vs I-II: OR 0.42, 95% CI 0.19–0.93). However, no obvious correlation was found with tumour size (≤ 5 cm vs > 5 cm: OR 1.35, 95% CI 0.83–2.18), tumour TNM stages (T3 & T4 vs T1 & T2: OR 1.21, 95% CI 0.76–1.92), lymphovascular invasion (LVI) (positive vs negative: OR 1.11, 95% CI 0.84–1.46), and HER-2 status (positive vs negative OR 2.51, 95% CI 0.84–7.51). Additionally, the heterogeneity was not obvious in the meta-analysis of tumour size, tumour TNM stages, lymphovascular invasion and PgR status (*I* ^2^ 0–25.8%).

**Table 4:** Meta-analysis of AGR2 overexpression and clinicopathological features of breast cancer.

**Stratification of breast cancer**	**NO. of study**	**References**	**Cases**	**Pooled OR (95% CI)**	***P*** **value**	**Heterogeneity**	
						***I*** ^**2**^ **(%)**	***p*** **value**
Tumor size ( > 5 cm **/** ≤ 5 cm)	2	Wu *et al*.^23^; Barraclough *et al*.^14^	464	1.35 (0.83–2.18)	0.213	0	0.809
Histological grade (III/I-II)	5	Fritzsche *et al*.^20^; Innes *et al*.^21^; Wu *et al*.^23^; Barraclough *et al*.^14^; Lacambra *et al*.^31^	1457	**0.42 (0.19–0.93)**	**0.032**	85.3	0.000
pT status (T3&T4/T1&T2)	3	Fritzsche *et al*.^20^; Wu *et al*.^23^; Lacambra *et al*.^31^	817	1.21 (0.76–1.92)	0.431	0	0.632
LVI(P/N)	4	Fritzsche *et al*.^20^; Innes *et al*.^21^; Wu *et al*.^23^; Lacambra *et al*.^31^	1042	1.11 (0.84–1.46)	0.469	25.8	0.257
ER status (P/N)	5	Fritzsche *et al*.^20^; Innes *et al*.^21^; Wu *et al*.^23^; Barraclough *et al*.^14^; Lacambra *et al*.^31^	1468	**4.08 (2.16–7.69)**	**0.000**	82.9	0.000
HER-2 status (P/N)	4	Fritzsche *et al*.^20^; Wu *et al*.^23^; Barraclough *et al*.^14^; Lacambra *et al*.^31^	1112	2.51 (0.84–7.51)	0.099	91.5	0.000
PgR status (P/N)	4	Innes *et al*.^21^; Wu *et al*.^23^; Barraclough *et al*.^14^; Lacambra *et al*.^31^	1110	**2.87 (2.17–3.80)**	**0.000**	0	0.702

Abbreviations: No.: number; OR: odds ratio; CI: confidence interval; LVI: lymphovascular invasion; P: positive; N: negative.

### Sensitivity and publication bias

**Figure 5:**
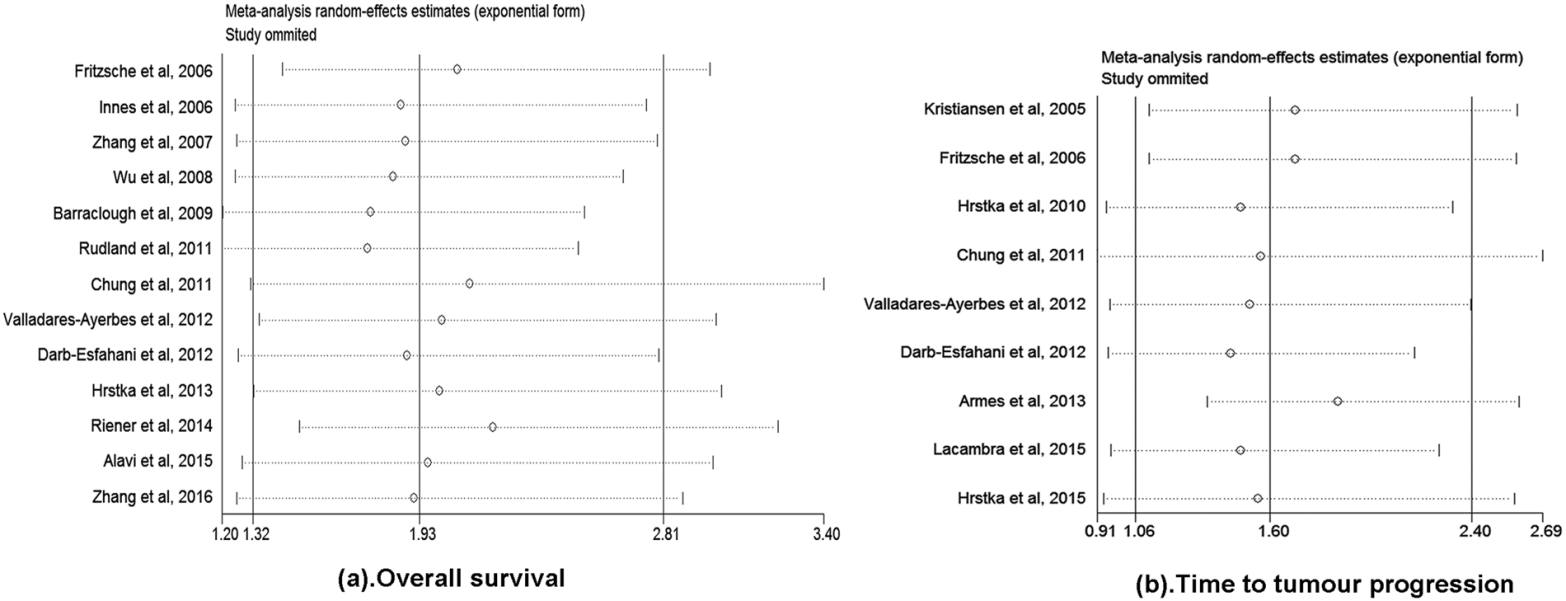
Sensitivity analysis of the meta-analysis. (**a**) Sensitivity analysis for the AGR2 overexpression with OS. (**b**) Sensitivity analysis for the AGR2 overexpression with TTP.

The sensitivity analyses were performed by omitting one study at a time to gauge the robustness of our results. We found that the pooled HRs was not significantly altered by excluding any single study, demonstrating that the results of this meta-analysis are statistically robust (Fig. 5). Furthermore, the publication bias in the included studies was assessed by combining the Begg’s funnel plot and Egger’s test. The results indicated no evidence for publication bias, as all *P* value for Begg’s test and Egger’s test were >0.05 (Fig. 4).

## Discussion

AGR2 mainly expressed in epithelial cells in human^34^. As a member of PDIs, AGR2 plays a pivotal role in maintaining endoplasmic reticulum homeostasis through regulating the unfolded protein response signalling (UPR)^35^. In addition, AGR2 has been implicated in a range of cell biological processes, in particular cellular transformation, cell migration and adhesion^36^. Moreover, AGR2 reportedly acts as a pro-oncogenic protein overexpressed in various cancers and involved in adenocarcinoma growth, cell metastasis^37,38^ and drug resistance^24^. Paradoxically, many studies found that elevated AGR2 levels did not predict the poor outcomes of solid tumour patients^9,19^. Thus, the prognostic value of AGR2 in tumours remains elusive and needs to be clarified.

In this article, we systematically evaluated AGR2 expression and the survival data of 3285 solid tumour patients from 20 different studies. Overall, our results demonstrated that AGR2 overexpression could predict poor OS (HR1.93, 95% CI 1.32–2.81) and poor TTP (HR1.60, 95% CI 1.06–2.40) of all solid tumour patients. These HR estimates were robust with no publication bias. However, high heterogeneity was observed across the studies included for this meta-analysis. The sensitivity analysis in this meta-analysis did not help to elucidate the source of heterogeneity. Therefore, we conducted subgroup analysis by study location and cancer types to address the source of heterogeneity. In subgroup analysis for breast cancer, both HR estimates for OS and TTP (OS: HR 3.02, 95% CI 1.03–8.81; TTP: HR 1.93, 95% CI 1.17–3.20) further indicates that AGR2 overexpression is predictive of poor prognosis in breast cancer patients. Meanwhile, the heterogeneity test showed no obvious heterogeneity for TTP (*I* ^2^ = 38.7%, *P* = 0.180), but it was still significant for OS (*I* ^2^ = 83.2%, *P* = 0.000). Further subgroup analysis was then performed and the results suggest that heterogeneity across the studies with sample ≥ 200 (*I* ^2^ = 57.3%, *P* = 0.216) or with follow-up time ≥ 80 months (*I* ^2^ = 50.2%, *P* = 0.134) can be effectively reduced, and the correlation of AGR2 overexpression with poor OS become significantly evident. Of note, subgroup analyses for the solid tumours group with breast cancer excluded showed that AGR2 overexpression was significantly associated with poor OS, but not with TTP. Thus, AGR2 might serve as a reliable prognostic marker for the OS of pooled patients with solid tumours.

We also analysed the relationship between AGR2 and clinicopathological features of breast cancer. Consistent with the previous study showing that AGR2 expression can be induced by estrogen in ERα expressing breast cancer cell lines^39^, the upregulation of AGR2 was found significantly correlated with positive ER and PgR status as well as low histological grade. As previously reported that AGR2 expression promoted cell lineage differentiation in murine stomach^40^, the co-expression of AGR2 and ER or PgR, and the association with the low histological grade indicated that AGR2 might be a marker of differentiation in breast cancer. However, in breast cancer patients, AGR2’s high expression was predictive of poor outcomes. Further functional studies are needed to clarify AGR2’s role in breast cancer.

Unlike its prognostic value in breast cancer, clinical effects of AGR2 expression on other tumour types remained inconclusive due to the existence of conflicting results^9,10,15^. Alavi *et al*. and Fritzsche *et al*. conducted cohort studies to explore the correlation of AGR2 expression status with the prognosis of lung cancer patients. AGR2 overexpression was found to contribute to the poor OS^15^. However, Fritzsche *et al*. found that AGR2 overexpression did not show any prognostic value in non-small-cell lung cancer (NSCLC)^9^. Interestingly, the other two studies focused on colorectal cancer and prostate cancer conducted by the same group (Fritzsche *et al*.) showed that in colorectal cancer AGR2’s up-regulation was strongly associated with improved OS compared with the control, while no prognostic value of AGR2 could be detected in prostate cancer^17,19^. Of note, in contrast to other selected studies, Fritzsche *et al*. reported a rather opposite observation that AGR2 overexpression predicted longer overall survival time of breast cancer patients^20^. We speculate that these discrepancies might be partially caused by differences in experimental protocols, antibody concentrations, and criteria for high AGR2 expression used in different research groups. Since further analyses could not be performed due to the insufficient number of existing studies, current data led us to a speculation that AGR2 might truly function as a tumour suppressor in some tumour types^19^, which however awaited future experimental verifications, especially in lung cancer, prostate cancer, ovarian cancer, gastric cancer, and colorectal cancer.

Alternatively, the different impact of AGR2 expression on solid tumours might be due to an apparently distinct regulation in certain cancer types. AGR2 is the human orthologue of the *Xenopus* Anterior Gradient-2 (XAG-2) protein^1^. In the *Xenopus* embryo, XAG-2 plays a key role in patterning the cement gland, a mucus-secreting tissue^41^. Similarly, AGR2 is predominately expressed in normal human colon, rectum, stomach, breast, and prostate^20^, which are the organs capable of secreting a variety of small molecules. Given that AGR2 was originally found in breast cancer specimen^42^, it is not surprising that almost half of the selected studies in this meta-analysis were breast cancer related. In normal mouse mammary glands, AGR2 is required for epithelial cell proliferation and lobuloalveolar development^39^. In breast cancer, AGR2 is co-expressed with estrogen receptor (ER) that directly regulates AGR2 expression^43^, and is significantly correlated with HER2 positive breast cancer^44^. Consistent with the previous studies, our study showed the co-expression of AGR2 and ER or PgR, and AGR2’s association with the low histological grade might indicate that AGR2 is a molecular marker of differentiation. This is also supported by the fact that AGR2 expression promoted cell lineage differentiation in murine stomach^40^. Intriguingly, it seems paradoxical to the prognosis of breast cancer patients for whom AGR2’ s high expression predicted poor outcomes. A possible explanation would be that AGR2 overexpression might increase breast cancer cells’ proliferative and invasive capacity. AGR2 overexpressed tumour cells was showed to have strong propensity for disseminating to lung^39^. Thus, we speculate that the mechanism for AGR2 overexpression predicting breast cancer’s poor prognosis could be: AGR2 expression was induced in an ER- or HER-dependent manner at the early stage of tumorigenesis, which led to treatment resistance and metastasis.

Some limitations of our study include: first, the standards for defining AGR2 positivity across the studies vary due to different experimental methods used for assessing AGR2 expression, which might lead to inter-study heterogeneity. Second, the sample size and the number of studies from certain cancer types, such as lung cancer, prostate cancer, ovarian cancer, gastric cancer and colorectal cancer, appear to be quantitatively insufficient. Third, as a secreted protein, the significant elevation of AGR2 in pancreatic juice from pancreatic cancer patients or in urine from prostate cancer patients suggests that AGR2 may also function extracellularly during the development of cancer^45,46^. In addition, both studies by Kyukwang Chung *et al*. and Valladares-Ayerbes *et al*. reported that the presence of detectable AGR2 in serum was significantly associated with the poor OS or DFS^25,29^. However, we could not perform a further analysis for the prognostic value of secreted AGR2, because only 2 studies observed serum AGR2 among the included studies. Although the mechanisms and the rate of AGR2 secretion from cytoplasm of tumour cells into the blood serum in different cancers might vary considerably^47^, we speculated that assessing AGR2 expression in tumour tissue along with a blood test for AGR2 protein may potentially lead to a more accurate and comprehensive assessment of the prognostic role of AGR2 in tumours.

In summary, despite the above limitations, our meta-analysis demonstrates that high AGR2 expression can serve as a prognostic predictor of OS for solid tumour patients, especially for breast cancer patients. Furthermore, in breast cancer, high expression of AGR2 trends to correlate with ER positivity, PgR positivity and low histological grade. However, further analyses and more trials on other types of cancers are required to confirm our conclusions.

## Methods

### Search strategy and selection criteria

This present meta-analysis was executed in accordance with the Preferred Reporting Items for Systematics Reviews and Meta-Analyses guidelines^48^.We performed a comprehensive literature search through the electronic databases PubMed, Embase, and Web of Science databases updated to January, 2017. We used the Medical Subject Heading (MeSH) terms and corresponding keywords to make the search strategy. The following combined search terms were used (“Anterior gradient 2“or HAG-2 or “Anterior gradient 2 homolog” or “Anterior gradient protein 2” or AGR2) AND (cancer or carcinoma or tumour or neoplasms). The references from selected articles were also examined by a hand search to find other relevant studies.

To be included in this meta-analysis, studies had to meet the following criteria: (1) The study evaluated the relationship between AGR2 expression and the prognosis of solid tumour patients, such as overall survival (OS) or disease-specific survival (DSS) or relapse-free survival (RFS) or disease-free survival (DFS) or progression-free survival (PFS) and clinicopathological features. (2) The study provided hazard ratios (HRs) with 95% confidence intervals (CIs) directly or these statistics could be calculated based on data presented. (3) A definite cut-off value to classify AGR2 expression as “positive” and “negative” or “high” and “low” was given. (4) Studies published in English or Chinese. (5) AGR2 expression was detected in tumour tissues or serum, rather than in cell lines. The exclusion criteria were as follows: (1) Reviews articles, letters, experimental studies, conference abstracts, case reports, duplicated publications or replicated samples. (2) Lacking sufficient data to estimate the HRs with 95% CIs. (3) Studies without OS or RFS or DFS or DSS or PFS for further quantification.

Two reviewers determined the eligibility of the screened studies independently. Discrepancies were solved by consensus after discussion.

### Data extraction and methodological quality assessment

Two authors independently extracted relevant information from each eligible study using a standardized data collection form. The following data were collected: types of cancer, name of first author, publication year, country, the number of patients, age median, detection method, location of AGR2 expression in tumour or serum, follow-up duration, outcome endpoints, and cut-off value to determine AGR2 positivity. When the prognosis was presented only as the Kaplan-Meier curves in some studies, the Engauge Digitizer V4.1 was then utilized to obtain the survival data, and Tierney’s method to calculate the HRs and 95%CIs^49^. The methodological quality of included studies was assessed by Newcastle-Ottawa Scale (NOS). The NOS consists of three quality parameters: selection (0–4 points), comparability (0–2 points), and outcome assessment (0–3 points). Studies with an NOS scores ≥ 6 were considered to be high-quality. Two reviewers performed the quality assessments separately.

### Statistical analysis

We applied the HRs with 95% CIs to evaluate the impact of AGR2 overexpression on outcomes of solid tumour patients in this meta-analysis. For analysing the association between AGR2 high expression and clinicopathological features, odds ratios (ORs) with 95% CIs were assessed. Begg’s funnel plot and Egger’s test determined the potential publication bias among selected studies. *P* values were two-sided and *P* < 0.05 was considered to be statistically significant. Sensitivity analysis was tested to examine the stability of the pooled results. Inter-study heterogeneity was quantified using Q-tests and I-squared test^50^. In the absence of significant heterogeneity (*P* > 0.10 or *I* ^2^ < 50%), a fixed effects model was appropriately used to calculate the pooled effect, otherwise the random effects model was employed. All statistical analyses were performed with Stata Version 12.0 (Stata Corporation, College Station, TX, USA).
